# Obesity Prevalence by Occupation in Washington State, Behavioral Risk Factor Surveillance System

**DOI:** 10.5888/pcd11.130219

**Published:** 2014-01-09

**Authors:** David K. Bonauto, Dayu Lu, Z. Joyce Fan

**Affiliations:** Author Affiliations: Dayu Lu, Z. Joyce Fan, Safety and Health Assessment and Research for Prevention Program, Washington State Department of Labor and Industries, Olympia, Washington.

## Abstract

**Introduction:**

Data that estimate the prevalence of and risk factors for worker obesity by occupation are generally unavailable and could inform the prioritization of workplace wellness programs. The aims of this study were to estimate the prevalence of obesity by occupation, examine the association of occupational physical activity and a range of health behaviors with obesity, and identify occupations in which workers are at high risk of obesity in Washington State.

**Methods:**

We conducted descriptive and multivariable analyses among 37,626 employed Washington State respondents using the Behavioral Risk Factor Surveillance System in odd numbered years, from 2003 through 2009. We estimated prevalence and prevalence ratios (PRs) by occupational groups adjusting for demographics, occupational physical activity level, smoking, fruit and vegetable consumption, and leisure-time physical activity (LPTA).

**Results:**

Overall obesity prevalence was 24.6% (95% confidence interval [CI], 24.0–25.1). Workers in protective services were 2.46 (95% CI, 1.72–3.50) times as likely to be obese as workers in health diagnosing occupations. Compared with their counterparts, workers who consumed adequate amounts of fruits and vegetables and had adequate LTPA were significantly less likely to be obese (PR = 0.91; 95% CI, 0.86–0.97 and PR = 0.63; 95% CI, 0.60–0.67, respectively). Workers with physically demanding occupational physical activity had a lower PR of obesity (PR = 0.83; 95% CI, 0.78–0.88) than those with nonphysically demanding occupational physical activity.

**Conclusion:**

Obesity prevalence and health risk behaviors vary substantially by occupation. Employers, policy makers, and health promotion practitioners can use our results to target and prioritize workplace obesity prevention and health behavior promotion programs.

## Introduction

Obesity has been declared an epidemic, posing a threat to public health. In the United States, the prevalence of obesity has increased dramatically in recent decades (1). In the workplace, obesity not only contributes to costs associated with sick leave and absenteeism but is also associated with increased risk of developing cancer, musculoskeletal disorders, cardiovascular disease, and stress (2). As the US workforce ages, these chronic diseases and their associated health care costs, which are predicted to be $4.2 trillion annually by 2023 (3), will place a tremendous burden on employers and the health care system.

On average, full-time workers spend more than 8 hours per day at work, with one-third to one-half of their workday spent sitting down (4). Occupational physical activity is a determinant of daily energy expenditure, and nonsedentary occupational physical activity may have a protective effect on the physical health of workers (5–8). Workers generally eat at least 1 meal during the workday, which makes the workplace a logical and valuable setting for promoting a healthy diet and the increase of physical activity. Studies support the effectiveness of workplace interventions that target improvement in obesity-related health behaviors of workers (9–11).

The Surgeon General’s Vision for a Healthy and Fit Nation includes recommendations for employers to create healthy worksites through promotion of physical activity and healthy eating in the workplace (12). Healthy People 2020 objectives include increasing the proportion of worksites that offer an employee health-promotion program, have nutrition or weight-management classes or counseling, and have exercise facilities and exercise programs (13). As a major component of health reform, the Prevention and Public Health Fund of the Affordable Care Act offers $200 million for wellness plan grants for small businesses (14). Yet, despite these strong public policy efforts to increase the health of the working population, few systematic measures exist of the health of the workforce by occupation. Estimating the prevalence of obesity by occupation can inform the development and evaluation of the effectiveness of work-based wellness programs. 

The objectives of this research were to 1) estimate the prevalence of obesity by occupation; 2) assess the association of obesity and occupation by considering the related factors of smoking, leisure-time physical activity (LTPA), adequate intake of fruits and vegetables, and occupational physical activity; and 3) identify occupations in need of workplace obesity prevention programs. This population-based study provides occupation specific analyses of health behaviors for consideration in the development of workplace wellness programs in Washington State and the nation.

## Methods

The Centers for Disease Control and Prevention’s Behavioral Risk Factor Surveillance System (BRFSS) is an annual, state-based, random-digit–dialed, landline telephone survey of the noninstitutionalized US civilian population aged 18 years or older that collects data on health conditions and behaviors. The BRFSS provides annual national and state-level prevalence estimates of obesity and biennial estimates of physical activity levels at work and during leisure time and assessment of healthy eating (15). Beginning in 2011, BRFSS included cellular telephone–only households in the sampling design and modified weighting methods; therefore, data preceding 2011 cannot be reliably compared with BRFSS data from 2011 to the present. We used data from the 2003, 2005, 2007, and 2009 Washington State BRFSS.

Since 2002, Washington State BRFSS has been collecting data on the occupation of currently employed respondents. For the survey respondents who were currently “employed for wages” or “self-employed,” interviewers asked the following questions for industries and occupation, respectively: “What kind of industries do you work in?” and “What is your job title?” If no job title was provided, the respondent was asked “What kind of work do you do?” The narrative text responses were coded to 1990 Census Industry and Occupation codes using the Standardized Occupation and Industry Coding (SOIC) software developed by the National Institute for Occupational Safety and Health (NIOSH). Industry coding was used to assist with the occupation coding. Those responses that were not coded via the SOIC program were manually assigned an occupation code by 3 NIOSH-trained coders who categorized responses into 3-digit occupational classifications on the basis of the National Center for Health Statistics instruction manuals (16) and SOIC (17). Manual coders were blinded to all other BRFSS response data. The 501 detailed occupational classes were further grouped into 28 broader occupational categories.

BRFSS demographic information included age, sex, race/ethnicity, educational level, and annual household income. Risk factors related to obesity included smoking status (collected annually), occupational physical activity levels, LTPA, and intake of fruits and vegetables (collected biennially in odd-numbered years).

Body mass index (BMI [kg/m^2^]) was calculated on the basis of respondents’ self-reported height and weight. A BMI of 30 or higher was considered obese. The 3 self-rated responses to occupational physical activity level were dichotomized to nonphysically demanding (response: “mostly sitting or standing”) and physically demanding work (responses: “mostly walking” or “mostly heavy labor or physically demanding work”). Data on vigorous LTPA (≥20 minutes/day for ≥3 times/week) and adequate daily intake of fruits and vegetables (≥5 servings of fruits and vegetables per day) were obtained as BRFSS calculated variables. Workers in the military (n = 208), in extraction occupations (n = 13), who were older than 65 years, or who had a BMI of less than 18.5 were excluded from the analyses. We estimated occupation-specific prevalence of obesity and multivariable prevalence ratios (PRs) for occupational groups, adjusting for occupational physical activity level, LTPA, smoking, and adequate fruit and vegetable consumption. Survey respondents provided oral informed consent for BRFSS participation. The Washington State institutional review board approved study protocol and content.

In this cross-sectional study, BRFSS calculates response and cooperation rates for each state using the Council of American Survey and Research Organizations guidelines (18). The Council of American Survey and Research Organizations is a survey research advocacy organization that provides a uniform definition and formula for measuring response and cooperation rates for survey research. The Washington State BRFSS survey response rates for the 2003, 2005, 2007, and 2009 surveys were 43.0%, 46.6%, 44.5%, and 48.2%, respectively; the cooperation rates for the same years were 67.7%, 74.2%, 70.1%, and 70.1%, respectively. We adjusted for survey year in the analyses. The data were weighted to account for the BRFSS sampling design. All analyses were performed using Stata software version 8.0 SE (StataCorp LP, College Station, Texas).

## Results

Of the 88,121 participants in the Washington State BRFSS during the 4 survey years, 45,878 (52.1%) were currently employed or self-employed at the time of survey administration. Of the 40,606 (46.1%) participants who reported occupation, 37,626 (42.7%) workers who had valid occupational codes, were aged 18 to 64 years, had a BMI of 18.5 or more, and were not working in military or extraction occupations were included in this study. The overall prevalence of obesity for all workers was 24.6% (95% confidence interval [CI], 24.0–25.1), ranging from 11.6% (95% CI, 8.0–15.2) for health diagnosing occupations (physicians, dentists, veterinarians, optometrists, and health diagnosing practitioners) to 38.6% (95% CI, 33.3–44.0) for truck drivers. Truck drivers and workers in transportation and material moving, protective services, and cleaning and building services had the highest prevalence of obesity (Table 1).

**Table 1:** Prevalence of Obesity by Demographic Characteristics and Occupation Among Workers, Washington State BRFSS, 2003, 2005, 2007, and 2009

Occupational Group (1990 Census Occupation Code)	n	Prevalence of Obesity, %^a^														
		Overall (95% CI)	Sex		Age			Annual Household Income, $			Education			Race/Ethnicity		
			Male	Female	18–29	30–44	45–64	< 35,000	35,000–74,999	≥ 75,000	< Grade 12	HS or some college	College Degree or Higher	White	Hispanic	Other
All occupations	37,626	24.6 (24.0–25.1)	25.1	23.9	18.3	25.1	27.2	26.2	26.9	22.3	24.1	28.0	20.5	24.7	26.2	22.7
Truck drivers (804)	545	38.6 (33.3–44.0)	38.9	36.3	—	38.9	42.9	37.1	41.4	40.0	35.8	38.2	48.5	39.8	—	—
Transportation and material moving (803; 806–859)	539	37.9 (31.9–43.9)	37.5	38.9	—	36.9	38.7	28.5	40.0	42.4	—	37.3	46.6	38.7	—	47.9
Protective services (413–427)	658	33.3 (28.5–38.1)	34.4	27.1	29.1	34.0	35.5	45.8	30.3	30.2	—	32.2	36.4	32.8	—	35.9
Cleaning and building services (448–455)	620	29.5 (24.7–34.3)	27.5	33.0	16.8	34.8	31.4	29.0	33.4	—	24.9	30.5	33.3	28.6	31.6	31.8
Health services (445–447)	1,089	28.8 (25.2–32.4)	24.9	30.2	25.6	32.4	28.2	31.0	30.7	18.7	40.1	30.1	20.3	28.9	23.2	34.8
Mechanics and repairers (503–549)	831	28.9 (25.0–32.8)	29.5	19.1	17.2	29.2	34.5	25.1	29.1	33.7	—	29.7	29.6	28.9	—	23.6
Administrative support, including clerical (303–389)	4,220	27.9 (26.1–29.7)	28.4	27.7	19.4	30.4	30.8	30.3	30.4	23.8	39.6	29.7	22.7	27.8	28.7	26.9
Personal services (403–407; 456–469)	1,028	27.2 (23.2–31.1)	25.6	27.5	30.8	25.1	26.0	28.5	27.6	20.9	43.2	29.0	12.0	24.4	32.1	40.2
Technicians and related support (203–235)	2,473	26.6 (24.4–28.8)	27.2	25.7	17.5	28.8	29.4	26.7	29.2	24.6	—	30.5	21.5	26.8	35.2	22.9
Precision production and plant operators (628–699)	960	26.1 (22.7–29.5)	26.3	25.1	19.7	27.6	26.9	25.0	26.0	26.5	16.7	26.7	27.1	28.6	—	14.9
Sales (243–285)	3,180	25.4 (23.4–27.4)	27.6	23.2	22.3	26.6	26.9	27.1	27.1	23.4	25.4	27.8	20.7	26.1	27.1	19.3
Management related (023–037)	2,183	25.1 (22.7–27.5)	25.8	24.7	22.8	23.9	27.1	30.3	26.5	23.3	—	28.5	22.3	25.0	42.8	18.9
Executive, administrative, and managerial (003–022)	4,893	24.4 (22.9–25.8)	24.9	23.6	15.7	23.7	26.8	25.4	27.4	23.5	18.3	29.3	21.3	24.4	21.3	25.6
Machine operators, assemblers, and inspectors (703–799)	587	23.9 (19.4–28.4)	23.7	25.0	19.2	19.7	31.4	22.5	27.1	15.5	26.9	24.2	17.5	22.1	27.7	—
Registered nurses (RNs) (095)	1,210	22.6 (19.8–25.5)	24.9	22.3	8.2	22.3	25.6	30.3	25.5	20.0	—	22.0	22.6	23.0	—	15.4
Farming, forestry, and fishing (473–499)	1,273	22.3 (18.8–25.8)	21.8	24.5	12.4	29.3	25.6	22.5	23.1	25.8	24.1	20.5	24.0	23.4	23.6	8.7
Teachers, excluding postsecondary (155–163)	3,008	21.8 (19.9–23.7)	26.9	19.6	13.8	20.9	25.1	24.5	22.7	19.8	—	27.8	20.3	21.5	32.6	21.9
Helpers, equipment cleaners, and laborers (864–889)	928	21.9 (18.4–25.3)	21.8	22.2	13.6	30.3	20.9	22.2	25.3	18.7	16.5	24.5	12.5	23.3	21.1	15.5
Mathematical and computer scientists (064–068)	465	21.8 (17.3–26.3)	21.3	23.2	—	18.1	28.9	—	32.4	20.3	—	30.1	17.7	23.8	—	14.2
Lawyers and judges (178–179)	271	21.7 (15.7–27.7)	19.8	19.5	12.6	20.0	21.7	22.9	22.5	15.2	—	26.6	16.5	19.1	—	24.9
Engineers, architects, and surveyors (043–063)	1,097	20.2 (17.4–23.0)	20.4	19.2	12.2	19.6	23.2	—	22.6	20.8	—	30.7	17.5	21.4	—	8.1
Food preparation and service (433–444)	1,111	20.1 (17.0–23.3)	18.3	21.9	14.8	23.3	30.6	22.8	17.9	24.1	13.7	22.0	18.0	20.3	19.1	20.5
Construction and construction trades (553–599)	1,307	19.9 (17.1–22.8)	19.9	18.0	10.8	20.7	26.3	16.5	21.2	21.5	22.3	19.7	18.7	19.3	23.7	21.8
Other professional specialties (164–165; 174–177; 183–199)	1,445	19.7 (17.2–22.3)	24.3	17.8	—	20.8	20.4	—	30.4	18.2	—	—	19.8	20.3	—	—
Health assessment and treating, excluding RNs (096–106)	391	18.2 (13.3–23.0)	24.2	14.8	—	19.8	17.6	—	20.6	17.9	—	25.7	17.1	19.0	—	—
Teacher, postsecondary (113–154)	330	17.6 (12.4–22.7)	18.0	16.6	—	19.7	16.6	—	26.8	13.0	—	—	17.4	16.0	—	—
Natural scientists and social scientists (069–083; 166–173)	523	17.3 (12.9–21.7)	20.0	13.6	—	11.1	23.0	—	17.7	15.6	—	24.8	16.7	18.2	—	—
Health diagnosing occupations (084–089)	461	11.6 (8.0–15.2)	11.9	11.3	—	8.4	15.5	—	16.6	11.0	—	—	9.8	12.4	—	10

Abbreviation: BRFSS, Behavioral Risk Factor Surveillance System; CI, confidence interval; HS, high school.

a Cells with an em dash (—) indicate inadequate sample size for prevalence estimate (n <50).

Table 2 presents the proportion of occupational physical activity level and health behavioral factors that were related to obesity by occupation. The proportion of current smokers was highest for truck drivers (34.1%; 95% CI, 28.6–39.4) and lowest for health assessment and treating occupations, excluding registered nurses (3.2%; 95% CI, 0.9–5.7). All health-care–related occupations exceeded 25.0% for intake of 5 or more servings of fruits and vegetables per day, with registered nurses exceeding all other occupations at 41.1% (95% CI, 37.5–44.6). Mechanics and repairers, mathematical and computer scientists, and truck drivers had the lowest proportion of adequate fruit and vegetable intake (Table 2). The highest proportion of vigorous LTPA was among workers in protective services (50.8%; 95% CI, 45.7–56.0), followed by health diagnosing occupations (45.3%; 95% CI, 39.6–50.9) and postsecondary teachers (42.7%; 95% CI, 35.8–49.6); machine operators, assemblers, and inspectors had the lowest proportion (27.2%; 95% CI, 22.4–31.9). Workers in health diagnosing occupations had consistently higher proportions of positive health behaviors than workers in any other occupations. More than 80% of workers in cleaning and building services, construction and construction trades, and farming, forestry, and fishing reported their work as physically demanding, but 3% or less of lawyers and judges and teachers, excluding postsecondary, did so (Table 2).

**Table 2:** Health Behaviors and Occupational Physical Activity of Workers, by Occupation, Washington State BRFSS, 2003, 2005, 2007, and 2009

Occupational Group (1990 Census Occupation Code)	No. of Respondents	% Current Smokers (95% CI)	% Adequate Fruit and Vegetable^a^ (95% CI)	% Vigorous LTPA^b^ (95% CI)	% Physically Demanding Occupational Activity^c^ (95% CI)
All occupations	37,626	17.2 (16.7–17.8)	23.3 (22.7–23.8)	34.3 (33.6–35.0)	35.8 (35.1–36.5)
Truck drivers (804)	545	34.1 (28.6–39.4)	15.8 (11.0–20.7)	30.6 (25.2–36.1)	41.7 (36.0–47.5)
Transportation and material moving (803; 806–859)	539	27.7 (22.2–33.5)	19.7 (15.1–24.2)	31.1 (25.7–36.5)	31.9 (26.0–37.8)
Protective services (413–427)	658	14.3 (10.7–18.0)	20.0 (15.8–24.1)	50.8 (45.7–56.0)	42.4 (37.2–47.5)
Cleaning and building services (448–455)	620	24.8 (20.3–29.4)	18.5 (14.4–22.6)	27.8 (22.7–32.8)	89.2 (85.8–92.7)
Health services (445–447)	1,089	25.8 (22.3–29.4)	25.3 (21.8–28.8)	28.3 (24.4–32.2)	59.0 (54.9–63.0)
Mechanics and repairers (503–549)	831	23.2 (19.5–26.9)	14.7 (11.6–17.7)	32.4 (28.3–36.4)	67.5 (63.5–71.5)
Administrative support, including clerical (303–389)	4,220	17.6 (16.1–19.2)	23.3 (21.5–25.0)	30.6 (28.6–32.7)	18.4 (16.7–20.1)
Personal services (403–407; 456–469)	1,028	20.9 (17.4–24.4)	29.6 (25.6–33.5)	31.8 (27.9–35.6)	46.9 (42.5–51.3)
Technicians and related support (203–235)	2,473	15.1 (13.3–16.8)	21.4 (19.4–23.4)	33.2 (30.8–35.6)	26.7 (24.5–29.0)
Precision production and plant operators (628–699)	960	25.6 (22.0–29.3)	19.8 (16.6–22.9)	29.0 (25.3–32.6)	49.4 (45.2–53.5)
Sales (243–285)	3,180	21.1 (19.2–23.1)	18.2 (16.4–20.1)	33.8 (31.5–36.2)	34.1 (31.8–36.4)
Management related (023–037)	2,183	13.4 (11.6–15.4)	22.5 (20.2–24.8)	33.8 (31.1–36.4)	10.4 (8.5–12.4)
Executive, administrative, and managerial (003–022)	4,893	12.6 (11.4–13.8)	24.6 (23.1–26.1)	34.6 (32.9–36.3)	20.8 (19.2–22.3)
Machine operators, assemblers, and inspectors (703–799)	587	27.1 (22.3–31.9)	17.3 (12.8–21.7)	27.2 (22.4–31.9)	54.3 (48.8–59.8)
Registered nurses (RNs) (095)	1,210	10.1 (7.9–12.2)	41.1 (37.5–44.6)	34.5 (31.1–38.0)	61.0 (57.5–64.5)
Farming, forestry, and fishing (473–499)	1,273	20.9 (17.4–24.4)	17.1 (14.1–20.0)	32.0 (27.6–36.5)	80.5 (77.6–83.5)
Teachers, excluding postsecondary (155–163)	465	7.0 (4.5–9.6)	27.0 (21.5–32.5)	37.4 (31.6–43.1)	2.1 (0.6–3.5)
Helpers, equipment cleaners, and laborers (864–889)	3,008	6.5 (5.2–7.6)	30.5 (28.5–32.6)	37.0 (34.8–39.3)	33.9 (31.7–36.1)
Mathematical and computer scientists (064–068)	928	27.4 (23.2–31.8)	15.0 (11.6–18.3)	29.4 (25.0–33.8)	71.8 (67.8–75.8)
Lawyers and judges (178–179)	271	7.3 (3.8–10.7)	25.9 (19.4–32.3)	40.1 (32.8–47.4)	3.0 (0.4–5.6)
Engineers, architects, and surveyors (043–063)	1,097	7.6 (5.7–9.6)	26.9 (23.8–30.1)	40.7 (37.1–44.3)	8.3 (6.4–10.2)
Food preparation and service (433–444)	1,111	32.8 (28.8–36.7)	20.6 (17.3–23.9)	37.4 (33.1–41.7)	59.5 (55.3–63.8)
Construction and construction trades (553–599)	1,307	30.8 (27.3–34.2)	17.3 (14.7–19.9)	34.7 (31.1–38.2)	84.7 (82.0–87.4)
Other professional specialties (164–165; 174–177; 183–199)	1,445	10.8 (8.8–12.8)	26.3 (23.5–29.1)	37.6 (34.4–40.8)	17.6 (15.0–20.1)
Health assessment and treating, excluding RNs (096–106)	391	3.2 (0.9–5.7)	37.5 (31.5–43.4)	36.8 (30.7–42.8)	34.9 (28.9–41.0)
Teachers, postsecondary (113–154)	330	3.3 (1.6–5.1)	30.8 (24.6–37.0)	42.7 (35.8–49.6)	8.0 (4.6–11.4)
Natural scientists and social scientists (069–083; 166–173)	523	7.3 (3.8–10.7)	30.4 (25.1–35.6)	37.9 (32.5–43.3)	18.1 (13.0–23.2)
Health diagnosing occupations (084–089)	461	3.7 (1.5–5.8)	38.2 (32.8–43.7)	45.3 (39.6–50.9)	23.0 (18.1–27.9)

Abbreviations: BRFSS, Behavioral Risk Factor Surveillance System; CI, confidential interval; LTPA, leisure-time physical activity.

a Defined as consuming 5 or more servings of fruit and vegetable per day.

b Defined as engaging in vigorous physical activity for 20 minutes or more per day, 3 or more times per week.

c The physically demanding occupational activity was determined by a respondent’s answer to the question, “When you are at work, which of the following best describes what you do?” Respondents who answered “mostly walking” or “mostly heavy labor or physically demanding work” were classified as having a physically demanding occupational activity.

Obesity was associated with certain demographic characteristics, socioeconomic factors, and health behaviors (Table 3). Prevalence ratios (PRs) for obesity were significantly higher among workers in older age groups than among workers aged 18 to 29, among male workers than among female workers, and among workers with less education than among workers with a college degree or higher. Compared with workers in the highest income group (≥$75,000), those in the lowest income group (<$35,000) had significantly higher obesity prevalence (PR = 1.09; 95% CI, 1.03–1.16), but the difference in PR was not significant for workers in the middle income group ($35, 000–$74,999). Race/ethnicity was not significantly associated with the prevalence of obesity. Nonsmokers were more likely to be obese (PR = 1.17; 95% CI, 1.09–1.25) than smokers. Workers who had adequate daily consumption of fruits and vegetables and adequate LTPA had significantly lower prevalence of obesity compared with those who consumed and exercised less (PR = 0.91; 95% CI, 0.86–0.97 and PR = 0.63; 95% CI, 0.60–0.67, respectively). Workers who characterized their occupational physical activity level as physically demanding had a lower prevalence of obesity (PR = 0.83; 95% CI, 0.78–0.88) than those who characterized their occupational physical activity level as nonphysically demanding (Table 3).

**Table 3:** Adjusted Prevalence Ratios of Obesity in Relation to Other Factors and Occupation Among Workers, Washington State BRFSS, 2003, 2005, 2007, and 2009

Factor or Occupational Group	Prevalence Ratio (95% CI)
**Age, y**	
18–29	1 [Reference]
30–44	1.38 (1.26–1.52)
45–64	1.46 (1.34–1.60)
**Sex**	
Female	1 [Reference]
Male	1.11 (1.05–1.17)
**Race/ethnicity**	
White	1 [Reference]
Hispanic	1.07 (0.96**–**1.20)
Other	0.94 (0.86**–**1.03)
**Annual household income, $**	
<35,000	1.09 (1.03–1.16)
35,000–74,999	1.07 (0.98**–**1.17)
≥75,000	1 [Reference]
**Educational attainment**	
Less than grade 12	1.34 (1.27–1.42)
High school or some college	1.25 (1.08–1.44)
College degree or higher	1 [Reference]
**Smoking**	
Current smoker	1 [Reference]
Not current smoker	1.17 (1.09–1.25)
**Vigorous leisure-time physical activity**	
<20 min/d and <3 times/wk	1 [Reference]
≥20 min/d and ≥3 times/wk	0.63 (0.60–0.67)
**Fruit and vegetable consumption, servings/d**	
<5	1 [Reference]
≥5	0.91 (0.86–0.97)
**Occupational physical activity^a^**	
Nonphysically demanding	1 [Reference]
Physically demanding	0.83 (0.78–0.88)
**Occupational group (1990 Census Occupational Code)^b^**	
Health diagnosing occupations (084–089)	1 [Reference]
Truck drivers (804)	2.45 (1.72–3.49)
Transportation and material moving (803; 806–859)	2.26 (1.56–3.28)
Protective services (413–427)	2.46 (1.72–3.50)
Cleaning and building services (448–455)	2.07 (1.43–2.99)
Health services (445–447)	2.03 (1.43–2.88)
Mechanics and repairers (503–549)	1.95 (1.37–2.79)
Administrative support, including clerical (303–389)	1.91 (1.37–2.65)
Personal services (403–407; 456–469)	1.93 (1.35–2.76)
Technicians and related support (203–235)	1.86 (1.34–2.60)
Precision production and plant operators (628–699)	1.71 (1.20–2.44)
Sales (243–285)	1.80 (1.29–2.50)
Management related (023–037)	1.75 (1.25–2.44)
Executive, administrative, and managerial (003–022)	1.75 (1.26–2.42)
Machine operators, assemblers, and inspectors (703–799)	1.49 (1.02–2.18)
Registered nurses (RNs) (095)	1.89 (1.34–2.67)
Farming, forestry, and fishing (473–499)	1.66 (1.15–2.38)
Teachers, excluding postsecondary (155–163)	1.73 (1.24–2.41)
Helpers, equipment cleaners, and laborers (864–889)	1.54 (1.07–2.21)
Mathematical and computer scientists (064–068)	1.65 (1.12–2.41)
Lawyers and judges (178–179)	1.54 (0.99–2.40)
Engineers, architects, and surveyors (043–063)	1.51 (1.06–2.14)
Food preparation and service (433–444)	1.67 (1.16–2.41)
Construction and construction trades (553–599)	1.42 (1.00–2.04)
Other professional specialties (164–165; 174–177; 183–199)	1.48 (1.05–2.09)
Health assessment and treating, excluding RNs (096–106)	1.58 (1.04–2.40)
Teachers, postsecondary (113–154)	1.36 (0.87–2.11)
Natural scientists and social scientists (069–083; 166–173)	1.32 (0.87–2.02)

Abbreviations: BRFSS, Behavioral Risk Factor Surveillance System; CI, confidence interval.

a Occupational activity was determined by a respondent’s answer to the question “When you are at work, which of the following best describes what you do?” Respondents who answered “mostly sitting or standing” were classified as having a nonphysically demanding occupational activity; respondents who answered “mostly walking” or “mostly heavy labor or physically demanding work” were classified as having a physically demanding occupational activity.

b The occupational group was adjusted for age, sex, race/ethnicity, annual household income, educational attainment, smoking, vigorous leisure-time physical activity, fruit and vegetable consumption, occupational physical activity, and survey year.

Among the 28 occupational groups, protective services (PR = 2.46; 95% CI, 1.72–3.50) and truck drivers (PR = 2.45; 95% CI, 1.72–3.49) had the highest risk of obesity, using the health diagnosing occupations as the reference group and after adjusting for multiple covariates (Table 3). PRs were not significant for natural and social scientists, postsecondary teachers, or lawyers and judges when compared with those of the health diagnosing occupations. Comparing with 2003, the prevalence of obesity among Washington workers increased in each study year; this increase in trend was independent of other obesity risk factors (data not shown).

## Discussion

We observed a significant disparity in obesity prevalence, intake of fruits and vegetables, and levels of LTPA across occupations in Washington State. We identified several occupations with significantly higher prevalence estimates of obesity than that of the referent occupation, health diagnosing occupations: truck drivers, transportation and material moving, protective services, and cleaning and building services. These findings are similar to the finding of another study that analyzed data of 603,139 US adult workers from 1986 to 2002 who responded to the National Health Interview Survey (NHIS). This study’s findings indicated that motor vehicle operators, workers in other transportations, workers in cleaning and building services, material-moving equipment operators, and workers in protective services had the highest obesity prevalence (19).

Generally, occupation is the crude aggregate measure of the demographic, social, and economic characteristics of worker populations. Previous research suggests that obesity prevalence varies by some of these same characteristics such as age, sex, race/ethnicity, annual household income, educational attainment, and LTPA (1,20,21). Male workers and workers in older age groups had higher PRs than female workers and those in younger age groups, similar to that of overall US adults (22). Another study using BRFSS data from 2006 through 2008 showed that Hispanics had 21% higher obesity rates than whites (23), but we did not observe a significant overall association between race/ethnicity and obesity. The population of Washington State workers is predominantly white, and estimates of obesity prevalence of nonwhite Washington workers by race are limited by the small number of respondents. We found that lower educational level and lower annual household income (<$35,000) were related to obesity prevalence. Workers with higher socioeconomic status, such as those in the health diagnosing occupations and postsecondary teachers, tended to have healthier behaviors than those with lower socioeconomic status. This finding may be explained by their higher educational attainment and income, which may correlate with higher expectations specific to personal health, appearance, and adequate economic capacity to purchase healthy but more expensive food items (24).

The importance of occupational physical activities in preventing obesity should be emphasized. Workers whose occupational physical activities were physically demanding, involving movement and heavy labor, had significantly lower prevalence of obesity compared with those with nonphysically demanding jobs. The Physical Activity Guidelines for Americans suggests at least 150 minutes per week of moderate-intensity or 75 minutes per week of vigorous-intensity aerobic physical activity for adults for substantial health benefits (25). According to a study by Bensley et al (26), occupational physical activities accounted for an additional 6.5% of the population meeting this recommendation. Our finding is consistent with a study of 10,785 workers from the Australian National Health Survey, which also found that workers with nonphysically demanding jobs were at significantly higher risk of being obese (6). Also, a recent analysis of 3,539 adults from the National Health and Nutrition Examination Survey suggested that having a high occupational physical activity level decreased the odds of abdominal obesity (5).

Employed smokers had lower obesity prevalence than employed nonsmokers, likely attributable to the appetite-suppressing effects of smoking (27). On average, 23.3% (95% CI, 22.7–23.8) and 34.3% (95% CI, 33.6–35.0) of Washington State adult workers met Healthy People 2010 recommended levels of fruit and vegetable consumption and LTPA level (Table 2), and our finding showed their significant protective effect on obesity (Table 3). In the Washington BRFSS data, occupations such as truck drivers and transportation and material moving appeared to have high PRs of obesity, a high proportion of smoking, and a low proportion of self-reported fruit and vegetable intake (Table 2). Although smoking has a protective effect on obesity (27), in our adjusted model, smoking did not offset the relationship between these occupational groups and obesity. Occupations such as truck drivers and transportation and material movers do not conform to employment in a single fixed worksite and are likely influenced by the availability of food choices in the broader environment, such as fast food and convenience stores (28). Systematic reviews of workplace nutrition and physical activity interventions suggest a modest benefit for promoting healthy diet among employees (29), achieving weight loss (9,11), and improving health behaviors (10). Our study suggests that societal, environmental, and occupational factors must be taken into account when developing workplace wellness programs for specific occupations. Moreover, previous research has shown significantly rising obesity rates among US workers from 1986 to 2002 regardless of race or sex (19). In our data, compared with 2003, the prevalence of obesity among Washington workers increased significantly in later years, but this increase in trend did not change the prevalence ratio for other obesity risk factors.

This study is subject to limitations. Because of the nature of a cross-sectional study, the relationship between occupation and obesity cannot provide evidence for causality. The BRFSS uses self-reported measures of height and weight rather than estimates of actual height and weight, which may lead to underestimation of BMI (30). Furthermore, BMI measures cannot distinguish between fat and lean tissue mass; workers with physically demanding jobs may be more physically fit and have a higher BMI because of increased muscle mass (31). For example, protective services (eg, firefighters, police officers) had a high prevalence of obesity but also had the highest proportion of vigorous LTPA (50.8%, 95% CI, 45.7–56.0). Because high physical standards for performance of job duties vary, there are possible biases across occupation related to BMI as a measure of obesity. Overestimates of obesity prevalence and possible biases in health behavioral measures may occur because of BRFSS data collection restrictions to landline telephones before 2011 and only to English- or Spanish-speaking respondents. Cellular telephone–only households were not included in the survey. There may be systematic errors in the automated and manual coding of occupation; however, if these errors occurred and whether they introduced bias into our results is unknown. To our knowledge, there is no previous state-level study that estimated occupation-specific obesity prevalence or that systematically examined risks of obesity by occupation while considering other related health behaviors among workers. To date, industry and occupation are not collected as part of the BRFSS core survey and are an elective module for states’ BRFSS coordinators to select. BRFSS is likely the optimal surveillance system for collecting data on state-level health-related behaviors by occupation because of the large sample size and the cost-effectiveness of such data collection.

Interventions to reduce obesity in the US population should be targeted to the workplace. According to the 2007 and 2008 BRFSS, the obesity prevalence among all workers was 26.1% in Washington State, similar to the national average of 27.0% (21), making the workplace a valuable opportunity to prevent obesity and promote health. Effective workplace health intervention programs will lower absenteeism and health care costs, improve health conditions and health behaviors of employees, and improve worker productivity. To meet the goals of Healthy People 2020 of increasing worksite health promotion programs, this study calls for promoting the systematic, ongoing collection of data on obesity and health-related behaviors by occupation. Characterizing obesity prevalence by occupation may influence key stakeholders to develop workplace wellness programs, which help identify unique societal and occupational factors for effective intervention to modify obesity risk factors. It also allows for the allocation of public health resources to high-risk worker groups that are most likely to benefit from such programs. In Washington State, allocation may best be directed toward the following occupational groups based on our findings: truck drivers and workers in transportation and material moving, protective services, and cleaning and building services. Evidence-based interventions suggest that employers identify the most effective interventions suitable to their specific workplace, promote worker engagement in physical activity with economic incentives, and introduce educational programs on healthier lifestyles, nutrition, and food budget management (11,14,19,26). Comprehensive workplace health intervention programs must be promoted and implemented to protect workers against the obesity epidemic in Washington State and in the United States.
